# Radiology Department: A Potential Source of Multidrug-Resistant Microorganisms: A Cross-Sectional Study at Tertiary Hospital, Palestine

**DOI:** 10.1155/2023/4441338

**Published:** 2023-12-18

**Authors:** Zena Odeh, Safaa Abatli, Mohammad Qadi

**Affiliations:** ^1^Department of Medical and Health Sciences, Faculty of Graduate Studies, An-Najah National University, P.O. Box. 7, Nablus, State of Palestine; ^2^Department of Radiology, An-Najah National University Hospital, Nablus, State of Palestine; ^3^Department of Biomedical Sciences, Faculty of Medicine and Health Sciences, An-Najah National University, P.O. Box. 7, Nablus, State of Palestine

## Abstract

**Introduction:**

Globally, healthcare facilities face a great challenge in the form of hospital-acquired infections (HAIs). Aside from the morbidity and mortality they cause, these illnesses are also extremely costly. Research on infection transmission in the medical field has been considerable, but not so much in the radiology department.

**Aim:**

This study aims to identify the presence of multidrug-resistant (MDR) microbes on surfaces that are frequently touched in computed tomography (CT), magnetic resonance imaging (MRI), ultrasound (US), plain X-ray examination rooms, and portable radiography that are susceptible to contamination as well as to investigate the potential dangers of contracting MDR organisms to patients and healthcare providers. *Materials and Method*. In this study, 160 swab samples were collected from the radiology department during May and June 2022. Samples were obtained from 80 predefined surfaces twice within and outside of CT and MRI examination rooms as well as from US and plain X-ray machines and portable X-ray machines. Samples were taken at 7:00 a.m. using cotton swabs following the regular cleaning procedure. Bacterial colony-forming units (CFUs) per square centimeter were calculated after swabbing a 100 cm^2^ surface.

**Results:**

Nearly all of the surfaces tested had bacterial CFUs. The highest contamination rate was found on keyboards ranging from (1.2–8) CFU/cm^2^, the sides of patient tables (1.2–20) CFU/cm^2^, knee coil (2.4–3) CFU/cm^2^, and patient leg supports (1.2–8) CFU/cm^2^. A noticeable increase in the contamination was noticed in June compared to May, and this was consistent with the increase in the number of isolated patients in the hospital, the workload in the radiology department, and the number of patients referred to the hospital. In our study, none of the examined sites showed contamination with MDR Gram-negative bacteria such as extended-spectrum beta-lactamases producing *Enterobacterales* (ESPL) or Carbapenemase-producing *Enterobacterales* (CPE). On the other hand, methicillin-resistant *Staphylococcus* (MRS), vancomycin-resistant *Staphylococcus* (VRS), and vancomycin-resistant *Enterococcus* (VRE) were detected.

**Conclusion:**

All of the radiology department equipment and sites could be a source of bacterial infection including MDR, so the obligatory and committed disinfection protocol must be revised and implemented in the morning and between patients.

## 1. Introduction

These days, the importance of infection control and prevention in health care centers cannot be overstated. Hospitals and health care centres often have surfaces contaminated with microorganisms, which can lead to serious healthcare-associated infections (HAIs) affecting both patients and health care workers. While it is well established that HAIa can cause serious diseases, there are still gaps in our understanding of how these bacteria are transmitted to patients. This gap underscores the critical importance of strict hygiene protocols and infection control measures within hospital and health care centre settings to reduce the risk of HAIs [1].

The radiology department and the use of different radiological equipment, including computed tomography (CT), magnetic resonance imaging (MRI), ultrasound (US), plain X-rays, and portable X-ray machines, have a vital role in diagnosing and monitoring different conditions [2, 3]. Cross-contamination is a critical concern in this area since patients are transferred to the radiology department from various clinics and departments with a wide spectrum of medical conditions. These patients' susceptibility to illness is already high due to their underlying medical conditions. Because these patients may come into direct or indirect contact with health care workers (HCWs) and contaminated surfaces and equipment, the risk of nosocomial infection transmission among them increases, making them vulnerable to HAIs [3, 4].

In recent years, the incidence of HAIs in radiology departments has witnessed a concerning increase. These infections jeopardize the safety of both HCWs and patients, further complicating radiographic investigation or intervention [5, 6].

HAIs are commonly referred to as nosocomial infections, which are defined as infections acquired within 48 hours of admission to a healthcare facility [6, 7]. In contrast to community-acquired infections (CAIs), these infections usually occur as a result of pathogens taking advantage of patients whose normal defenses against infection are compromised [3]. Extensive literature has highlighted the Centres for Disease Control and Prevention's (CDC) classification of nosocomial infection sites into 13 types with up to 50 infection sites [8]. These infections put hospitalized patients at a big risk, leading to significant morbidity and mortality, particularly in low-income countries rather than in high-income countries around the world [4, 7, 9].

High-touch surfaces in patient rooms, such as bed controls, bed rallies, call buttons, and bedside tray tables, represent a critically important multidrug-resistant organism (MDRO) reservoir and increase the risk of acquisition by other patients, visitors, and hospital staff who are exposed to them. The presence of MDR bacteria within hospital settings poses a significant threat that extends to the selection of empiric antibiotics that target MDR bacteria. This can perpetuate a vicious cycle of increasing antimicrobial resistance [2, 10, 11].

Several studies have demonstrated that disinfection plays a significant role in controlling microbial carriage in people who are not harboring MDROs. Beyond disinfection, there are several essential infection prevention methods, such as environmental cleaning, hand hygiene, contact precautions, and active screening. Disinfection offers a universal solution by safeguarding both MDRO carriers and noncarriers [12].

Disinfection is becoming increasingly significant in most hospitals because the number of patients harboring MDROs asymptomatically is growing over time [13]. Increased research on MDROs persistence in the hospital environment and subsequent transmission has recently resulted in a greater emphasis on hospital environmental hygiene [14–16].

Drug-resistant and MDROs include methicillin-resistant *Staphylococcus aureus* (MRSA), methicillin-resistant *Staphylococcus epidermis* (MRSE), vancomycin-resistant *Staphylococcus aureus* (VRSA), vancomycin-resistant *Enterococcus* (VRE), extended-spectrum beta-lactamases producing *Enterobacterales* (ESPL), carbapenemase-producing *Enterobacterales* (CPE), carbapenem-resistant *Acinetobacter baumannii* (CR-AB), and carbapenem-resistant *Pseudomonas aeruginosa* (CR-PA) [5, 16, 17].

In a recent study, cleaning surfaces by using a sodium lauryl sulfate-based detergent can prevent MRSA transmission in health-care settings and reduce the risk of surface contamination at hospitals in general and radiology departments in particular [18]. In a previous investigation involving bacterial detection, samples were obtained from CT equipment, and the results indicated that the CT wrap was the most contaminated with germs, prompting the development of a novel cleaning procedure [6].

The real pandemic outbreak of COVID-19, which killed several million people and had major global economic consequences, is an indication that much more work is necessary to tackle infectious diseases and the growing global issue of antibiotic resistance [19]. In the case of hospitalized patients, the case-fatality rate linked with bacteremia ranges from 35% to 50% and is usually associated with MDR Gram-negative bacteria such as ESBL and CRE [20].

Globally, prevalent carbapenemases are found in *Enterobacteriaceae,* including *Klebsiella pneumoniae* carbapenemases (KPC) [21]. *K. pneumoniae,* which produces KPC, is a worldwide threat [22]. In a recent study, bacterial contamination was found in almost all selected areas in the radiology department, including MRI and CT equipment; fortunately, no MDROs such as MRSA, ESPL, or CPE were detected [7].

The spread of MDROs and HAIs presents a major challenge to the global healthcare community especially developing countries, as they have much higher risks of HCAIs with a ratio of 20 : 1 as compared to developed countries [23].

This cross-sectional study was conducted at a tertiary hospital in Palestine, where we investigated the presence of multidrug-resistant organisms on highly touched surfaces in the radiology department as well as the potential danger of patients and healthcare workers contracting multidrug-resistant organisms. Swab samples were taken from the commonly hand-touched sites in the department of radiology and cultured at a microbiology lab. Antimicrobial resistance was tested.

## 2. Materials and Methods

### 2.1. Study Design

A cross-sectional study design was carried over at the radiology department at Tertiary Hospital, Palestine.

### 2.2. Study Population

The commonly hand-touched sites were assessed according to the previous studies [7], and an adapted flow chart was created. Briefly, swab samples targeted the surfaces inside and outside: plain X-ray, CT, MRI, US, and portable X-ray instruments, in addition to examination rooms, as shown in Table 1.

### 2.3. Study Time

The sample swabs were collected in two separate time periods: the first one was in May 2022 and the other one was in June 2022. The same sites were investigated, and these sites are the commonly hand-touched sites surface described in Table 1.

### 2.4. Study Sample and Settings

160 swab samples were collected as described in Table 1 to cover the 80 determined commonly hand-touched sites in May and June. Each swab sample covered an area of 100 cm^2^ (around 10 cm *∗* 10 cm). Samples were taken in each month, every other day.

Sampling was carried out with a sterile swab. The sterile swab was presoaked in sterile normal saline and inserted directly after swabbing into a falcon tube with 2 ml phosphate buffer saline (PBS) and transported immediately to the microbiology lab at the Faculty of Medicine and Health Sciences at An-Najah National University.

### 2.5. Bacterial Culture and Detection

Each tube was vortexed for one minute, and 100 *µ*l were cultured into each of the following agar plates:

1. Blood agar.
2. Chocolate agar.
3. MacConkey agar.
4. Mannitol salt agar (MSA).
5. MSA + Oxacillin (6 *µ*g/ml)⟶Methicillin-resistant *Staphylococcus* (MRS) detection.
6. MacConkey agar + Meropenem (1 *µ*g/ml)⟶ Carbapenem-resistant *Enterobacteriaceae* (CRE) detection.
7. MacConkey agar + Cefotaxime (1 *µ*g/ml)⟶ Extended Spectrum Beta Lactamase (ESBL) detection.
8. Bile esculin + Vancomycin (6 *µ*g/ml)⟶ Vancomycin-resistant *Enterococcus* (VRE) detection.

Antibiotics used in this study were purchased from Sigma Aldrich, while the media were purchased from Oxoid. Media with antibiotics were prepared as described in the Clinical and Laboratory Standard Institute (CLSI) 2021 and as described in the literature [24, 25].

All plates were incubated at 37 degree/24 hours aerobically, while chocolate agar plates were incubated in 5% CO_2._

American Type Culture Collection (ATCC) strains (*E. coli* ATCC 25922 *and S. aureus* ATCC 25923) and clinically confirmed strains (MRSA, ESBL, CRE and VRE) were used as controls for the prepared media in each preparation.

Finally, all the identified multidrug-resistant isolates were confirmed as such through antibiotic susceptibility testing, following the CLSI protocols.

### 2.6. Vancomycin Resistance

The screening method was used to detect vancomycin resistance. In short, bile esculin media was prepared with a concentration of 6 *µ*g/ml; further confirmation was approached through *E*-test. *E* test was applied for those strains that needed to be screened for vancomycin sensitivity: MRS detected using MSA + oxacillin and colonies grown on bile esculin agar with vancomycin.

### 2.7. Contamination Rate Calculation

Simply put each commonly encountered site in our study was swabbed with a sterile, premoistened PBS swab, targeting a 100 cm^2^ (10 cm *∗* 10 cm) area. After swabbing, the swab was placed in an already prepared falcon tube containing 2 ml sterile PBS, vortexed well, and 100 *µ*l was transferred and cultivated on the prepared media's surface.

Following the incubation period, colonies on each plate were counted, and the findings were given as CFU/cm^2^, taking the swabbed area and volume of PBS buffer in the tubes into account (calculation CFU/cm^2^: counted colonies number *∗* 0.2), as shown in Table S3.

### 2.8. Ethical Approval

Ethical approval was taken from the institutional review board (IRB) at An-Najah National University and An-Najah National University Hospital.

## 3. Results

### 3.1. Bacterial Detection

Bacterial growth was nearly detected in all targeted sites. Regarding samples collected in May, bacterial growth was detected in 49/80 sites. Samples collected in June showed growth in 52/80 sites. Interestingly, in total, 60/80 sites showed bacterial growth as shown in Tables S1 and S2.

In the radiology department as well as all other inanimate objects, Gram-positive bacteria are predominant in and out of CT, MRI, plain X-ray, US rooms, and portable X-ray, while Gram-negative was only detected in four sites, as shown in Tables S1 and S2.

### 3.2. Total Bacterial Contamination

Regarding bacterial contamination ≥1 CFUs/cm^2^, in May, out of 80 sites examined, 21 sites (26%) had contamination, while in June, 20 sites (25%) were considered contaminated. In total, 29 sites showed contamination of ≥1 CFUs/cm^2^, as shown in Table 2.

### 3.3. Gram-Positive: *Staphylococcus* (MSA-Growth)

Concerning contamination with bacteria that can grow on MSA which is mainly *Staphylococcus*, in May, out of 80 sites examined, 9 sites had a contamination rate of ≥1 CFUs/cm^2^, while in June, 18 sites were considered contaminated. In total, 19 sites showed contamination of ≥1 CFUs/cm^2^, as shown in Table 3.

### 3.4. Gram-Positive: MRS

Regarding the surface contamination with MRS, six sites in May and 13 sites in June showed growth on MSA + oxacillin, which means MRS is suspected to be present. Later on, all suspected colonies were confirmed as MRS after subculture, and cefoxitin resistance was demonstrated by the disk diffusion method. Out of 13 sites, only one site had a contamination rate ≥1 CFUs/cm^2^ in June and none in May. However, the site with a contamination rate ≥1 CFUs/cm^2^ is the centre of the patient table of the CT Canon with 1.4 CFUs/cm^2^, as shown in Table 4.

### 3.5. Gram-Positive: Vancomycin-Resistant *Staphylococcus* (VRS)

After MRS detected and confirmed, all MRS isolates were tested for vancomycin sensitivity using E-test. In May, no VRS was detected, and all isolates were found to be vancomycin sensitive *Staphylococcus*. Surprisingly, in June, 7 out of 13 detected MRS were confirmed as VRS, namely, samples from head pillow of CT Canon, centre of patient table CT Canon, right side gantry control panel CT Canon, primax X-ray portable touch screen, patient table tools in US for general use room, keyboard in control room of US for general use, and left side of MRI patient Table 2.

### 3.6. Gram-Positive: VRE

VRE were detected in five of the examined sites, one site in May with a contamination rate <1 CFUs/cm^2^, while the other four times were in June, with a contamination rate of ≥1 CFUs/cm^2^, as shown in Table 5.

### 3.7. Gram-Negatives

Our results showed that only four Gram-negative bacteria were isolated from four different sites during the two cohorts done in May and June 2022. Three of these contaminated sites have contamination rate of <1 CFUs/cm^2^ during May, while the fourth contaminated site (centre of patient table–CT cannon) has a contamination rate of 1.4 CFUs/cm^2^ and this contamination was detected in June, as shown in Tables S1, S2 and 6.

### 3.8. Gram-Negative: ESBL and CRE

None of the equipment sites from the radiology department that were tested showed growth of ESBL and CRE, according to the data in Tables S1 and S2.

### 3.9. Contamination Rate

Because of the increasing number of patients and an increased number of referred patients in June, the contamination rate average of 1.3 CFUs/cm^2^ is greater than in May 0.79 CFUs/cm^2^, as shown in S1 and S2 Tables, while for the study period (May and June), no differences were noticed in the disinfectant materials used to disinfect the surfaces, the time, and frequency at which the surfaces were cleaned.

Growth conditions affect the contamination rate, as the data shown in Table S3, the contamination rate on chocolate agar was higher than the contamination rate on blood agar for almost all sites during the two cohorts. Chocolate agar has a higher contamination rate than blood agar mostly because chocolate agar contains lysed red blood cells with better growth for fastidious organisms and due to the fact that chocolate agar plates were incubated in an anaerobic environment [26].

The results showed a contamination median value greater than 3 CFU/cm^2^ from seven common surface sites tested in the CT, MRI, US, and plain X-ray; centre and sides of the examination table X-ray patient's tables, knee coils, MRI patient's leg support, and all of the radiology machine keyboards.

Alarming findings reveal that the highest contamination rate was found in the CT Canon's core of the patient table and the large touch screen of the US for general use, with 10 CFUs/cm^2^ and 12.8 CFUs/cm^2^, respectively. Another alarming piece of data shows the high contamination rate on the right and left side of MRI patient (Table 2) MRI patient leg support 2, keyboard in the X-ray control room, left side of patient table of CT Canon, CT Siemen's keyboard in the control room, and keyboard and table of US for general use; in both cohorts, as shown in Table S3.

## 4. Discussions

The findings of our study indicate that Gram-positive bacteria were more detected in the radiology department than Gram-negative ones, and this was expected. This result is consistent with previous investigations that have found that Gram-positive were more common than Gram-negative bacteria on inanimate surfaces in the radiology department [7]. It was demonstrated that Gram-positive bacteria have a stronger potential for surviving on inanimate surfaces and environment [27]. Furthermore, Gram-positive bacteria also make up a significant portion of the skin's microbiota [28]. In short, the higher occurrence of Gram-positive bacteria in the radiology department can be attributed to their survival characteristics and their presence on the skin.

In our study, the MRSA contamination rate on the inert surface was relatively high 28/160 (17.5%). On the other hand, in Sweden, swabs were taken from the bore, table, and wrap of two quaternary care inpatient CT scanners; the wrap was the most contaminated item on a CT scanner, and the prevalence of MRSA was significantly low [29]. However, in another study in Ireland, from 125 samples collected from the radiology department, MRSA was detected from one sample only, bore in the MRI gantry [30].

However, in earlier research on cassettes and lead aprons carried out in radiology departments across the United Kingdom, there was no evidence of MRSA [2]. In our research, no MRS was detected from the MRI gantry, but the other 27 samples taken from different sites of the radiology department's equipment were positive for MRS. MRSA was present on an X-ray cassette that had been utilized in the operation room [31]. In addition, our investigation showed no evidence of MRS and bacterial contamination on the X-ray cassette.

In general, the sample area makes up only a small portion of the overall surfaces, which may reduce the sensitivity of the test when attempting to identify resistant bacteria that are present in low numbers. In addition, the purpose of this investigation was to identify any contamination on the surfaces inside and outside the radiology examination rooms and equipment and determine whether they were more likely to be contaminated. In the majority of the CT, X-ray, and US examination rooms both inside and outside, as well as on the patient tables of the MRI machines, the keyboards contained a noticeable bacterial contamination rate that ranged 1–8 CFU/cm^2^.

This has additionally been shown to be the case in other research, as they showed that work station sites in the radiology department are 64.3% (9 of 14) contaminated with *S. aureus* and 21.4% (3 of 14) are contaminated with enteric organisms [7, 32]. It is probable that this is because medical staff members do not adequately disinfect their hands after dealing with the patient within the examination room or that they do not regularly disinfect the keyboards and patient tables. Both of these factors contribute to the spread of infection. There has been a lot of research on how important it is to practice good hand hygiene in order to prevent the spread of infection [33].

The simplest, most effective, and least expensive strategy to prevent the spread of microorganisms is to practice strict hand cleanliness. In our study, a large number of CFU/cm^2^ was found in most cases on the LT side of MRI patient (Table 2) the centre of patient table CT CANON, and the large touch screen for US for general use with 8.4, 10, and 12.8 CFUs/cm^2^; respectively. One possible explanation is that the patients' clothing had been in contact with their bodies for at least 15 minutes and HCWs hands when dealing with patients during examination. In addition, the examination tables' sides of MRI, CT, and X-ray had contamination rates ranging from 1–8.4 CFU/cm^2^. This may be due to the fact that patients sit on the examination table with their skin in close contact with the side of the examination table. The examination table's sides and centre, as well as the MRI knee coil and patient legs support, are most likely not thoroughly disinfected. In general, a large number of surfaces showed bacterial contamination that was higher than the permissible limit of one CFU/cm^2^. Meanwhile, other studies showed low numbers of CFUs/cm^2^ on the side of the tunnel of the MRI camera in the radiology department [7] and a decrease in bacterial growth presence in the magnetic field [34, 35].

The surfaces may not have been cleaned thoroughly enough, and this may be a cause of the infection. This could be the result of, for instance, an insufficient amount of staff education on infection management, or it could be because the cleaning is not effective. When deciding on a cleaning procedure, there are many factors to take into consideration. It should be efficient, but at the same time, it should not have any negative effects on either humans or the environment, and it should not be too expensive [36].

Alterations to cleaning procedures and the kind of materials used have varying effects on certain infections. A cleaning solution containing hydrogen peroxide is excellent against bacteria and viruses, but it is harmful to humans and cannot be used for continuous cleaning [37].

Self-disinfecting surfaces covered with copper and silver have also been studied, and this has been demonstrated to minimize HAI. For the pathogen, efficiency, the environment, and the economy to all benefit from disinfection procedures, further research is needed [38].

In the radiology department, fortunately, we detected a low number of CFU/cm^2^ approximately near zero CFU/cm^2^ on the side of the Siemens CT gantry, patient table of X-ray, head coil MRI, surface coils of MRI, and probes for interventional ultrasonography compared to other sites in the MRI examination room, the side of the X-ray patient table, and probes of US for general use. Since patients contact this location practically every time they use these machines, it is highly unlikely that it is disinfected more regularly than other parts of the apparatus.

Concerning the effect that magnetic fields have on the number of bacteria present, additional research needs to be carried out. A previous study investigated the potential effect of a rotating magnetic field against bacteria through cell wall disruption and changes in morphology [39].

We were able to detect substantial differences in the contamination between months of May and June in the radiology department. In June, patient isolation, workload, and the number of referrals for patients were all higher than in May. As a reflection of that, the average contamination rate from all sites in June was 1.3 CFUs/cm^2^, while in May, it was 0.79 CFUs/cm^2^. Moreover, six sites showed contamination with MRS in May compared to 13 sites in June, with only one site in June having a contamination rate of ≥1 CFUs/cm^2^. Surprisingly, 7 sites showed contamination with VRS, and all were in June. Regarding contamination with Gram-negative bacteria, a contamination rate of ≥1 CFUs/cm^2^ was detected only at one site in June. In addition to that, VRE was detected at one site in May with a contamination rate of <1 CFUs/cm^2^, while it was detected at four sites in June, with a contamination rate of ≥1 CFUs/cm^2^.

Based on our results, we can say that reducing contamination effectively in a healthcare setting, such as a radiology department, requires a comprehensive approach involving staff education, proper cleaning and disinfection protocols, and ongoing monitoring. We emphasize that hospital staff must be educated and trained regularly on proper hand hygiene techniques. We also propose implementing a regular cleaning schedule for all surfaces within the radiology department to ensure that cleaning includes high-touch areas like doorknobs, keyboards, phones, and equipment surfaces [40–43].

Another technique for infection control is the use of disposable barriers (e.g., plastic covers) on equipment surfaces that come into direct contact with patients. These can be changed between each patient, reducing the risk of cross-contamination [44].

Because patients and family members are a significant part of the contamination equation, we encourage educating patients about the importance of hand hygiene and covering coughs and sneezes to reduce the risk of spreading infections within the department [45].

We also encourage conducting regular audits and assessments of cleaning and infection control practices to ensure that the whole team has a clear understanding of the hand hygiene importance and infection prevention precautions [46, 47].

### 4.1. Limitations

In general, it is important to note that the sampled area in our study represents only a fraction of the entire surfaces being studied at a specific period of time. This limitation could potentially reduce the test's sensitivity and may restrict the generalizability of our results, especially when trying to detect resistant bacteria that may be present in small quantities.

Our study observes significant variability in contamination rates between different months, which raises questions about the consistency of factors affecting contamination levels. Factors such as patient workload, isolation practices, and referral rates can fluctuate and complicate the interpretation of results.

## 5. Conclusion

There is an ongoing debate all around the world regarding whether or not the setting of a hospital contributes to the spread of HAIs. However, there is evidence from research that supports the concept that hospitals can operate as a crucial reservoir of numerous nosocomial infections in a variety of environments. These environments include surfaces, medical equipment, and water systems.

In this investigation, it was concluded that

1. Radiology department could be a source of healthcare-acquired infection. Gram-positive bacteria were the most present bacteria and multidrug-resistant bacteria were detected from various sites with a contamination rate which exceeded the limit of 1 CFU/cm^2^ for bacterial contamination.
2. The relatively high methicillin-resistant *Staphylococcus* contamination rate observed in this study highlights the importance of regular monitoring for *Staphylococcus* contamination in radiology departments. It emphasizes the need for stringent infection control practices to prevent the spread of methicillin-resistant *Staphylococcus* within healthcare facilities.
3. Increase in the work load, referred and isolated patients which were proportional to the increase in the contamination rate, the presence of Gram-negative, and multidrug-resistant bacteria.
4. Surface cleaning and disinfecting must frequently focus on keyboards in the radiology department, examination patient table sides and centres, knee coil, US machine, and patient legs support in particular. Proper hand hygiene can also help reduce the risk of bacterial transmission within examination rooms.

In summary, our research provides practical insights that can guide infection control practices in radiology departments. It highlights the need for rigorous cleaning and disinfection protocols, awareness of specific bacterial strains like methicillin-resistant *Staphylococcus*, and further exploration of innovative solutions to minimize bacterial contamination and reduce the risk of infections in healthcare settings.

## Figures and Tables

**Table 1:** Commonly hand-touched sites.

MRI	CT CANON	CT SEMENS	Interventional US	US for general use	X-ray	Portable X-ray primax	Portable X-ray care stream
Canter of patient Table 1	Head pillow	Head support	Linear probe	Linear probe	X-ray cassette	X-ray cassette	X-ray cassette
Head support	RT side edge of patient table	RT side edge of patient table	Curve linear probe	Curve linear probe	Wall Bucky control panel	Touch screen	Touch screen
Head coil 1	Centre of patient table	Centre of patient table	Key board of US	US keyboard	Wall Bucky	Hands of machine	Hand of machine
Anaesthesia machine	Injector control panel	Another table in the CT room	Touch screen of US	Small touch screen	Touch screen of X-ray tube		
RT side gantry control panel	CT keyboard	Leg support		Table in control room	Prep medication table		
X-ray cassette	RT side gantry control panel	RT side gantry control panel		Door of examination room	Centre of patient table		
	LT side gantry control panel	LT side gantry control panel		Table of US tools	RT side edge of patient table		
Centre of gantry	Centre of gantry	Centre of gantry		Hand of US	LT side edge of patient table		
Ear support (headphone)	Surface of emergency trolley	Prep medication table		Head pillow	Key board in control room		
Surface coil 1	Control room keyboard	Keyboard in control room		Mouse in the control room	Mouse		
RT side edge of patient Table 1	LT side edge of the patient table	LT side edge of the patient table		Keyboard in control room	X-ray keyboard		
Surface coil 2	Injector touch screen	Another table in the CT room		Large touch screen			
Head coil 2	CT mouse	CT mouse		Patient table			
Knee coil	Mouse in the control room						
Centre of patient Table 2							
RT side edge of patient Table 2							
LT side edge of patient Table 2							
Leg support 1							
Leg support 2							

**Table 2:** Sites with the contamination rate of ≥1 CFU/cm^2^, either on blood or on chocolate agar.

Sample number	Sample source	May experiment		June experiment	
		Contamination rate chocolate agar	Contamination rate blood agar	Contamination rate chocolate agar	Contamination rate blood agar
2	MRI head support	0.6	2	0.4	0.2
4	Head pillow of CT CANON	0.6	0	2.2	0.4
6	Centre of patient table CT CANON	1.6	0.4	8.8	10
11	Keyboard of CT CANON	2	0.6	6	4
14	LT side gantry control panel CT CANON	0.4	1	0	0
15	Primax X-ray portable touch screen	0	0.6	1	6
25	Keyboard of US for general use	1.2	1.4	2.4	1.2
30	RT side of MRI patient table	4.8	1	0	0
33	Keyboard in CT CANON control room	3	0	0.6	1.6
34	CT Siemen's keyboard control room	4	8	6	6
44	Keyboard of interventional US	1	0.6	0.2	0
48	Patient leg support of CT semen's	0.2	0	0.6	1.2
51	Large touch screen for US for general use	0	0	12.8	0.4
52	Table of US for general use in control room	4.8	1.8	1.6	1.4
56	Hand of US for general use	1	0.2	0.8	0.4
58	Mouse of US for general use in control room	2.2	0.8	0.4	0.2
59	Keyboard in control room of US for general use	3.4	0.6	1	0.2
60	RT side of X-ray patient table	0.2	0	2.4	0.8
63	Hand of carestearm portable	8	0.2	0	0.8
64	Carestream touchscreen portable	0.2	0.2	5.2	5
66	LT side of patient table CT CANON	8	8	1.6	1.2
67	Touch screen of injecter in CT CANON control room	0.2	0	1.8	1.6
72	Knee coil	2.4	3	0.6	0.4
73	Centre of MRI patient Table 2	1.4	0	2.8	2.4
74	RT side of MRI patient Table 2	2.8	4.8	3	0.6
75	LT side of MRI patient Table 2	5.8	4.2	8.4	6
77	Keyboard of X-ray in control room	3.2	3.4	6	8
79	Patient leg support 1	2.2	2.4	0.4	0.4
80	Patient leg support 2	2	3.4	8	5.4

**Table 3:** Sites contaminated with bacteria that can grow on MSA agar during two months.

Sample number/month	Sample source	Mannitol salt agar (CFUs/cm^2^)
4/May and June	Head pillow of CT CANON	0.2/0.4
5/June	RT side of patient table CT CANON	0.2
6/May and June	Centre of patient table CT CANON	1.6/4
7/May	Head coil MRI 1	0.2
11/June	Keyboard of CT CANON	3.4
14/May	LT side gantry control panel CT CANON	0.2
15/June	Primax X-ray portable touch screen	4
24/May and June	Table of prep medication in X-ray room	0.2/0.2
25/May and June	Keyboard of US for general use	1/1.4
26/May	Patient table of US for general use	0.2
27/June	Ear plugs of MRI	0.8
29/June	MRI surface coil 1	0.2
30/May	RT side of MRI patient table	2.2
31/June	LT side of MRI patient Table 1	3
32/June	Trolley emergency in CT CANON room	0.4
33/June	Keyboard in CT CANON control room	1.8
34/June	CT Siemens keyboard control room	6
35/June	CT Siemens mouse control room	0.4
36/June	CT Siemens inside gantry	0.2
38/June	RT side edge of CT patient table	0.2
39/June	CT Siemens head support	0.6
44/May	Keyboard of interventional US	0.4
45/May	Touch screen of interventional US	0.2
48/May and June	Patient leg support of CT Siemens	0.6/0.2
49/June	Linear probe for US general use	1.2
50/June	Curve linear probe for US for general use	0.4
52/May and June	Table of US for general use in control room	1.8/1.6
54/May and June	Small touch screen for US general use	0.2/0.2
55/May and June	Patient table tools in US for general use room	0.6/0.4
56/May and June	Hand of US machine for general use	0.6/1
57/June	Patient head pillow in US for general use room	0.6
58/May	Mouse of US for general use in control room	0.6
59/May and June	Keyboard in control room of US for general use	1.6/1
60/June	RT side of X-ray patient table	0.8
62/June	Keyboard in control room of X-ray	0.4
63/June	Hand of Carestream portable	0.2
64/June	Carestream touch screen portable	0.2
66/May and June	LT side of patient table CT CANON	3.6/1.4
67/June	Touch screen of injector in CT CANON control room	1.6
72/June	Knee coil	0.8
73/June	Centre of MRI patient Table 2	1.2
74/May and June	RT side of MRI patient Table 2	2.2/1.6
75/May and June	LT side of MRI patient Table 2	1.2/4.4
77/May and June	Keyboard of X-ray in control room	0.6/5.6
78/June	Mouse of X-ray in control room	0.8
79/May and June	Patient leg support MRI 1	1.2/0.4
80/May and June	Patient leg support MRI 2	0.2/5.4

**Table 4:** Sites contaminated with MRS on mannitol salt agar with oxacillin.

Sample number/month	Sample source	Mannitol salt agar with oxacillin (CFUs/cm^2^)
4/June	Head pillow of CT CANON	0.4
5/June	RT side of patient table CT CANON	0.2
6/May and June	Centre of patient table CT CANON	0.2/1.4
11/June	Keyboard of CT CANON	0.2
15/June	Primax X-ray portable touch screen	0.4
34/May	CT Siemens keyboard control room	0.4
55/June	Patient table tools in US for general use room	0.4
59/June	Keyboard in control room of US for general use	0.2
66/May and June	LT side of patient table CT CANON	0.6/0.6
72/May	Knee coil	0.2
74/May	RT side of MRI patient Table 2	0.2
75/June	LT side of MRI patient Table 2	0.6
79/May	Patient leg support 1	0.4

**Table 5:** Sites harbors VRE in the radiology department, on bile esculin agar with vancomycin.

Sample number/month	Sample source	Bile esculin agar with vancomycin (CFUs/cm^2^)
4/June	Head pillow of CT CANON	6
6/June	Centre of patient table CT CANON	8
15/June	Primax X-ray portable touch screen	2
26/May	Patient table of US for general use	0.2
67/June	Touch screen of injector in CT CANON control room	3

**Table 6:** Samples with growth of Gram-negative bacteria.

Sample number/month	Sample source	CFU/cm^2^
6/June	Centre of patient table CT CANON	1.4
7/May	Head coil MRI 1	0.2
27/May	Ear plug of MRI	0.2
75/May	LT side of MRI patient Table 2	0.2

## Data Availability

Data used in this study will be available from the corresponding author upon reasonable request.

## Supplementary Materials

Supplementary Materials Table S1: sample source and number of colonies on each plate during May. Table S2: sample source and number of colonies on each plate during June. Table S3: calculated contamination rate (CFUs/cm^2^) during the two months on both blood and chocolate agar from each sample source.
