# Supplementary material for: Radiology Department: A Potential Source of Multidrug-Resistant Microorganisms: A Cross-Sectional Study at Tertiary Hospital, Palestine
Source: Can J Infect Dis Med Microbiol. 2023 Dec 18;2023:4441338. doi: 10.1155/2023/4441338 (PMC10749721; doi:10.1155/2023/4441338)
Supplement: Supplementary Materials — Table S1: sample source and number of colonies on each plate during May. Table S2: sample source and number of colonies on each plate during June. Table S3: calculated contamination rate (CFUs/cm2) during the two months on both blood and chocolate agar from each sample source. [file 4441338.f1.docx]

**Table S1**: Shows sample source and number of colonies on each plate during May.

| Bile esculine Agar with vancomycin | Manetol Salt Agar with oxacilline | macConky with cefotaxim | macConky with meropenem | Manetol Salt Agar | macConky Agar | Chocolate Agar | Blood Agar | sample source | Experiment 1: May |
| --- | --- | --- | --- | --- | --- | --- | --- | --- | --- |
| 0 | 0 | 0 | 0 | 0 | 0 | 3 | 1 | Center of patient table MRI 1 | 1 |
| 0 | 0 | 0 | 0 | 0 | 0 | 3 | 10 | MRI head support | 2 |
| 0 | 0 | 0 | 0 | 0 | 0 | 0 | 0 | Wall Bucky | 3 |
| 0 | 0 | 0 | 0 | 1 | 0 | 3 | 0 | Head pillow of CT CANON | 4 |
| 0 | 0 | 0 | 0 | 0 | 0 | 0 | 0 | RT side of patient table CT CANON | 5 |
| 0 | 1 | 0 | 0 | 8 | 0 | 8 | 2 | Center of patient table CT CANON | 6 |
| 0 | 0 | 0 | 0 | 1 | 1 | 1 | 1 | Head coil MRI 1 | 7 |
| 0 | 0 | 0 | 0 | 0 | 0 | 0 | 0 | Control panel for contrast media injector | 8 |
| 0 | 0 | 0 | 0 | 0 | 0 | 0 | 0 | Wall Bucky control panel | 9 |
| 0 | 0 | 0 | 0 | 0 | 0 | 0 | 0 | X-Ray cassette | 10 |
| 0 | 0 | 0 | 0 | 0 | 0 | 10 | 3 | Keyboard of CT CANON | 11 |
| 0 | 0 | 0 | 0 | 0 | 0 | 0 | 0 | RT side gantry control panel CT CANON | 12 |
| 0 | 0 | 0 | 0 | 0 | 0 | 0 | 0 | X-Ray cassette of primax portable | 13 |
| 0 | 0 | 0 | 0 | 1 | 0 | 2 | 5 | LT side gantry control panel CT CANON | 14 |
| 0 | 0 | 0 | 0 | 0 | 0 | 0 | 3 | primax X-Ray portable touch screen | 15 |
| 0 | 0 | 0 | 0 | 0 | 0 | 0 | 0 | CT CANON gantry | 16 |
| 0 | 0 | 0 | 0 | 0 | 0 | 0 | 0 | Hand of primax X-Ray portable | 17 |
| 0 | 0 | 0 | 0 | 0 | 0 | 2 | 1 | Anasthesia Machine | 18 |
| 0 | 0 | 0 | 0 | 0 | 0 | 1 | 0 | RT side of control panel of MRI gantry | 19 |
| 0 | 0 | 0 | 0 | 0 | 0 | 0 | 0 | Table of MRI control Room | 20 |
| 0 | 0 | 0 | 0 | 0 | 0 | 0 | 0 | LT side of control panel MRI gantry | 21 |
| 0 | 0 | 0 | 0 | 0 | 0 | 0 | 0 | touch screen of X-Ray tube | 22 |
| 0 | 0 | 0 | 0 | 0 | 0 | 0 | 0 | inside gantry of MRI | 23 |
| 0 | 0 | 0 | 0 | 1 | 0 | 0 | 0 | Table of prep medication in X-Ray room | 24 |
| 0 | 0 | 0 | 0 | 5 | 0 | 6 | 7 | Keyboard of US for general use | 25 |
| 1 no black colony | 0 | 0 | 0 | 1 | 0 | 1 | 0 | Patient table od US for general use | 26 |
| 0 | 0 | 0 | 0 | 0 | **1** | 1 | 1 | ear plugs of MRI | 27 |
| 0 | 0 | 0 | 0 | 0 | 0 | 0 | 0 | Center of patient table X-Ray | 28 |
| 0 | 0 | 0 | 0 | 0 | 0 | 1 | 0 | MRI surface coil 1 | 29 |
| 0 | 0 | 0 | 0 | 11 | 0 | 24 | 5 | RT side of MRI patient table | 30 |
| 0 | 0 | 0 | 0 | 0 | 0 | 2 | 3 | LT side of MRI patient table 1 | 31 |
| 0 | 0 | 0 | 0 | 0 | 0 | 2 | 0 | Trolley emergency in CT CANON room | 32 |
| 0 | 0 | 0 | 0 | 0 | 0 | 15 | 0 | Keyboard in CT CANON control room | 33 |
| 0 | 2 | 0 | 0 | 0 | 0 | 20 | 40 | CT Siemen’s Keyboard control room | 34 |
| 0 | 0 | 0 | 0 | 0 | 0 | 4 | 2 | CT Siemen’s mouse control room | 35 |
| 0 | 0 | 0 | 0 | 0 | 0 | 0 | 0 | CT Siemen’s inside gantry | 36 |
| 0 | 0 | 0 | 0 | 0 | 0 | 0 | 0 | CT Siemen’s center of patient table | 37 |
| 0 | 0 | 0 | 0 | 0 | 0 | 1 | 0 | RT side edge of CT Siemen’s Patient table | 38 |
| 0 | 0 | 0 | 0 | 0 | 0 | 4 | 3 | CT Siemen’s head support | 39 |
| 0 | 0 | 0 | 0 | 0 | 0 | 0 | 0 | RT side CT semen’s gantry control panel | 40 |
| 0 | 0 | 0 | 0 | 0 | 0 | 0 | 0 | LT side CT semen’s gantry control panel | 41 |
| 0 | 0 | 0 | 0 | 0 | 0 | 0 | 0 | linear probe for interventional US | 42 |
| 0 | 0 | 0 | 0 | 0 | 0 | 0 | 0 | Curve linear probe for interventional US | 43 |
| 0 | 0 | 0 | 0 | 2 | 0 | 5 | 3 | Keyboard of interventional US | 44 |
| 0 | 0 | 0 | 0 | 1 | 0 | 0 | 1 | Touch screen of interventional US | 45 |
| 0 | 0 | 0 | 0 | 0 | 0 | 0 | 0 | Table of prep medication in CT semen’s room | 46 |
| 0 | 0 | 0 | 0 | 0 | 0 | 1 | 0 | Trolley emergency in CT semen’s room | 47 |
| 0 | 0 | 0 | 0 | 3 | 0 | 1 | 0 | Patient leg support of CT semen’s | 48 |
| 0 | 0 | 0 | 0 | 0 | 0 | 0 | 0 | Linear probe for US general use | 49 |
| 0 | 0 | 0 | 0 | 0 | 0 | 1 | 0 | Curve linear probe for US for general use | 50 |
| 0 | 0 | 0 | 0 | 0 | 0 | 0 | 0 | Large touch screen for US for general use | 51 |
| 0 | 0 | 0 | 0 | 9 | 0 | 24 | 9 | Table of US for general use in control room | 52 |
| 0 | 0 | 0 | 0 | 0 | 0 | 0 | 0 | Door of US for general use examination room | 53 |
| 0 | 0 | 0 | 0 | 1 | 0 | 1 | 0 | Small touch screen for US general use | 54 |
| 0 | 0 | 0 | 0 | 3 | 0 | 4 | 1 | Patient table tools in US for general use room | 55 |
| 0 | 0 | 0 | 0 | 3 | 0 | 5 | 1 | Hand of US for general use | 56 |
| 0 | 0 | 0 | 0 | 0 | 0 | 3 | 0 | patient Head pillow in US for general use room | 57 |
| 0 | 0 | 0 | 0 | 3 | 0 | 11 | 4 | Mouse of US for general use in control room | 58 |
| 0 | 0 | 0 | 0 | 8 | 0 | 17 | 3 | Keyboard in control room of US for general use | 59 |
| 0 | 0 | 0 | 0 | 0 | 0 | 1 | 0 | RT side of X-Ray patient table | 60 |
| 0 | 0 | 0 | 0 | 0 | 0 | 0 | 0 | LT side of X-Ray patient table | 61 |
| 0 | 0 | 0 | 0 | 0 | 0 | 0 | 2 | Keyboard in control room od X-Ray | 62 |
| 0 | 0 | 0 | 0 | 0 | 0 | 40 | 1 | Hand of carestearm portable | 63 |
| 0 | 0 | 0 | 0 | 0 | 0 | 1 | 1 | carestream touchscreen portable | 64 |
| 0 | 0 | 0 | 0 | 0 | 0 | 0 | 0 | X-Ray cassette of carestream portable | 65 |
| 0 | 3 | 0 | 0 | 18 | 0 | 40 | 40 | LT side of patient table CT CANON | 66 |
| 0 | 0 | 0 | 0 | 0 | 0 | 1 | 0 | Touch screen of injector in CT CANON control room | 67 |
| 0 | 0 | 0 | 0 | 0 | 0 | 0 | 1 | Mouse of CT CANON | 68 |
| 0 | 0 | 0 | 0 | 0 | 0 | 0 | 0 | Mouse in CT CANON control room | 69 |
| 0 | 0 | 0 | 0 | 0 | 0 | 0 | 0 | Surface coil MRI 2 | 70 |
| 0 | 0 | 0 | 0 | 0 | 0 | 0 | 0 | Head coil MRI 2 | 71 |
| 0 | 1 | 0 | 0 | 0 | 0 | 12 | 15 | Knee coil | 72 |
| 0 | 0 | 0 | 0 | 0 | 0 | 7 | 0 | Center of MRI patient table 2 | 73 |
| 0 | 1 | 0 | 0 | 11 |  | 14 | 24 | RT side of MRI patient table 2 | 74 |
| 0 | 0 | 0 | 0 | 6 | 1 | 29 | 21 | LT side of MRI patient table 2 | 75 |
| 0 | 0 | 0 | 0 | 0 | 0 | 4 | 0 | LT side of CT semen’s patient table | 76 |
| 0 | 0 | 0 | 0 | 3 | 0 | 16 | 17 | Keyboard of X-Ray in control room | 77 |
| 0 | 0 | 0 | 0 |  | 0 | 0 | 0 | Mouse of X-Ray in control room | 78 |
| 0 | 2 | 0 | 0 | 6 | 0 | 11 | 12 | Patient leg support MRI 1 | 79 |
| 0 | 0 | 0 | 0 | 1 | 0 | 10 | 17 | Patient leg support 2 MRI | 80 |

**Table S2:** Shows sample source and number of colonies on each plate during June.

| Bile esculine Agar with vancomycin | Manetol Salt Agar with oxacilline | macConky with cefotaxim | macConky with Meropenem | Manetol Salt Agar | macConky Agar | chocolate Agar | Blood Agar | Sample Source | Experiment 2: June |
| --- | --- | --- | --- | --- | --- | --- | --- | --- | --- |
| 0 | 0 | 0 | 0 | 0 | **0** | 0 | 0 | Center of patient table MRI 1 | 1 |
| 0 | 0 | 0 | 0 | 0 | **0** | 2 | 1 | MRI head support | 2 |
| 0 | 0 | 0 | 0 | 0 | **0** | 0 | 0 | Wall Bucky | 3 |
| 30 | 2 | 0 | 0 | 2 | **0** | 11 | 2 | Head pillow of CT CANON | 4 |
| 0 | 1 | 0 | 0 | 1 | **0** | 2 | 2 | RT side of patient table CT CANON | 5 |
| 40 | 7 | 0 | 0 | 20 | 7 | 44 | 50 | center of patient table CT CANON | 6 |
| 0 | 0 | 0 | 0 | 0 | 0 | 0 | 0 | Head coil MRI 1 | 7 |
| 0 | 0 | 0 | 0 | 0 | 0 | 0 | 0 | control panel for contrast media injector | 8 |
| 0 | 0 | 0 | 0 | 0 | 0 | 0 | 0 | wall Bucky control panel | 9 |
| 0 | 0 | 0 | 0 | 0 | 0 | 0 | 1 | X-Ray cassette | 10 |
| 0 | 1 | 0 | 0 | 17 | 0 | 30 | 20 | Keyboard of CT CANON | 11 |
| 0 | 0 | 0 | 0 | 0 | 0 | 0 | 0 | RT side gantry control panel CT CANON | 12 |
| 0 | 0 | 0 | 0 | 0 | 0 | 0 | 0 | X-Ray cassette of primax portable | 13 |
| 0 | 0 | 0 | 0 | 0 | 0 | 0 | 0 | LT side gantry control panel CT CANON | 14 |
| 10 | 2 | 0 | 0 | 20 | 0 | 5 | 30 | primax X-Ray portable touch screen | 15 |
| 0 | 0 | 0 | 0 | 0 | 0 | 0 | 0 | CT CANON gantry | 16 |
| 0 | 0 | 0 | 0 | 0 | 0 | 0 | 0 | hand of primax X-Ray portable | 17 |
| 0 | 0 | 0 | 0 | 0 | 0 | 0 | 0 | Anesthesia Machine | 18 |
| 0 | 0 | 0 | 0 | 0 | 0 | 0 | 0 | RT side of control panel of MRI gantry | 19 |
| 0 | 0 | 0 | 0 | 0 | 0 | 4 | 4 | Table of MRI control Room | 20 |
| 0 | 0 | 0 | 0 | 0 | 0 | 0 | 0 | LT side of control panel MRI gantry | 21 |
| 0 | 0 | 0 | 0 | 0 | 0 | 0 | 0 | touch screen of X-Ray tube | 22 |
| 0 | 0 | 0 | 0 | 0 | 0 | 0 | 0 | inside gantry of MRI | 23 |
| 0 | 0 | 0 | 0 | 1 | 0 | 4 | 1 | Table of prep medication in X-Ray room | 24 |
| 0 | 0 | 0 | 0 | 7 | 0 | 12 | 6 | Keyboard of US for general use | 25 |
| 0 | 0 | 0 | 0 | 0 | 0 | 0 | 0 | Patient table od US for general use | 26 |
| 0 | 0 | 0 | 0 | 4 | 0 | 4 | 2 | ear plugs of MRI | 27 |
| 0 | 0 | 0 | 0 | 0 | 0 | 1 | 0 | center of patient table X-Ray | 28 |
| 0 | 0 | 0 | 0 | 1 | 0 | 1 | 1 | MRI surface coil 1 | 29 |
| 0 | 0 | 0 | 0 | 0 | 0 | 0 | 0 | RT side of MRI patient table | 30 |
| 0 | 0 | 0 | 0 | 60 | 0 | 100 | 100 | LT side of MRI patient table 1 | 31 |
| 0 | 0 | 0 | 0 | 2 | 0 | 4 | 2 | Trolley emergency in CT CANON room | 32 |
| 0 | 0 | 0 | 0 | 9 | 0 | 3 | 8 | keyboard in CT CANON control room | 33 |
| 0 | 0 | 0 | 0 | 30 | 0 | 30 | 30 | CT Siemens Keyboard control room | 34 |
| 0 | 0 | 0 | 0 | 2 | 0 | 3 | 2 | CT Siemens mouse control room | 35 |
| 0 | 0 | 0 | 0 | 1 | 0 | 0 | 0 | CT Siemens inside gantry | 36 |
| 0 | 0 | 0 | 0 | 0 | 0 | 0 | 2 | CT Siemens center of patient table | 37 |
| 0 | 0 | 0 | 0 | 1 | 0 | 3 | 2 | RT side edge of CT Siemens Patient table | 38 |
| 0 | 0 | 0 | 0 | 3 | 0 | 0 | 4 | CT Siemens head support | 39 |
| 0 | 0 | 0 | 0 | 0 | 0 | 0 | 0 | RT side CT Siemens gantry control panel | 40 |
| 0 | 0 | 0 | 0 | 0 | 0 | 0 | 0 | LT side CT Siemens gantry control panel | 41 |
| 0 | 0 | 0 | 0 | 0 | 0 | 0 | 0 | linear probe for interventional US | 42 |
| 0 | 0 | 0 | 0 | 0 | 0 | 0 | 0 | Curve linear probe for interventional US | 43 |
| 0 | 0 | 0 | 0 | 0 | 0 | 1 | 0 | Keyboard of interventional US | 44 |
| 0 | 0 | 0 | 0 | 0 | 0 | 1 | 0 | Touch screen of interventional US | 45 |
| 0 | 0 | 0 | 0 | 0 | 0 | 0 | 0 | Table of prep medication in CT Siemens room | 46 |
| 0 | 0 | 0 | 0 | 0 | 0 | 0 | 0 | Trolley emergency in CT Siemens room | 47 |
| 0 | 0 | 0 | 0 | 1 | 0 | 3 | 6 | Patient leg support of CT Siemens | 48 |
| 0 | 0 | 0 | 0 | 6 | 0 | 14 | 3 | Linear probe for US general use | 49 |
| 0 | 0 | 0 | 0 | 2 | 0 | 2 | 2 | Curve linear probe for US for general use | 50 |
| 0 | 0 | 0 | 0 | 0 | 0 | 64 | 2 | Large touch screen for US for general use | 51 |
| 0 | 0 | 0 | 0 | 8 | 0 | 8 | 7 | Table of US for general use in control room | 52 |
| 0 | 0 | 0 | 0 | 0 | 0 | 0 | 0 | Door of US for general use examination room | 53 |
| 0 | 0 | 0 | 0 | 1 | 0 | 1 | 1 | Small touch screen for US general use | 54 |
| 0 | 2 | 0 | 0 | 2 | 0 | 3 | 2 | Patient table tools in US for general use room | 55 |
| 0 | 0 | 0 | 0 | 5 | 0 | 4 | 2 | hand of US for general use | 56 |
| 0 | 0 | 0 | 0 | 3 | 0 | 0 | 3 | patient Head pillow in US for general use room | 57 |
| 0 | 0 | 0 | 0 | 0 | 0 | 2 | 1 | Mouse of US for general use in control room | 58 |
| 0 | 1 | 0 | 0 | 5 | 0 | 5 | 1 | keyboard in control room of US for general use | 59 |
| 0 | 0 | 0 | 0 | 4 | 0 | 12 | 4 | RT side of X-Ray patient table | 60 |
| 0 | 0 | 0 | 0 | 0 | 0 | 1 | 2 | LT side of X-Ray patient table | 61 |
| 0 | 0 | 0 | 0 | 2 | 0 | 1 | 5 | Keyboard in control room od X-Ray | 62 |
| 0 | 0 | 0 | 0 | 1 | 0 | 0 | 4 | hand of carestearm portable | 63 |
| 0 | 0 | 0 | 0 | 2 | 0 | 26 | 25 | carestream touchscreen portable | 64 |
| 0 | 0 | 0 | 0 | 0 | 0 | 2 | 0 | X-Ray cassette of carestream portable | 65 |
| 0 | 3 | 0 | 0 | 7 | 0 | 8 | 6 | LT side of patient table CT CANON | 66 |
| 15 | 0 | 0 | 0 | 8 | 0 | 9 | 8 | touch screen of injector in CT CANON control room | 67 |
| 0 | 0 | 0 | 0 | 0 | 0 | 0 | 3 | mouse of CT CANON | 68 |
| 0 | 0 | 0 | 0 | 0 | 0 | 0 | 0 | mouse in CT CANON control room | 69 |
| 0 | 0 | 0 | 0 | 0 | 0 | 0 | 0 | surface coil MRI 2 | 70 |
| 0 | 0 | 0 | 0 | 0 | 0 | 0 | 0 | Head coil MRI 2 | 71 |
| 0 | 0 | 0 | 0 | 4 | 0 | 3 | 2 | Knee coil | 72 |
| 0 | 0 | 0 | 0 | 6 | 0 | 14 | 12 | Center of MRI patient table 2 | 73 |
| 0 | 0 | 0 | 0 | 8 | 0 | 15 | 3 | RT side of MRI patient table 2 | 74 |
| 0 | 3 | 0 | 0 | 22 | 0 | 42 | 30 | LT side of MRI patient table 2 | 75 |
| 0 | 0 | 0 | 0 | 0 | 0 | 4 | 0 | LT side of CT Siemens patient table | 76 |
| 0 | 0 | 0 | 0 | 28 | 0 | 30 | 40 | Keyboard of X-Ray in control room | 77 |
| 0 | 0 | 0 | 0 | 4 | 0 | 5 | 4 | Mouse of X-Ray in control room | 78 |
| 0 | 0 | 0 | 0 | 2 | 0 | 2 | 2 | patient leg support 1 | 79 |
| 0 | 0 | 0 | 0 | 27 | 0 | 40 | 27 | leg support 2 MRI | 80 |

**Table S3:** Calculated contamination rate (CFUs/cm^2^) during the two months on both Blood and Chocolate agar from each sample source.

|  |  | May- Experiment | | June-Experiment | |
| --- | --- | --- | --- | --- | --- |
| Sample Number | Sample Source | Contamination rate chocolate agar | Contamination rate blood agar | Contamination rate chocolate agar | Contamination rate blood agar |
| 1 | Center of patient table MRI 1 | 0.6 | 0.2 | 0 | 0 |
| 2 | MRI head support | 0.6 | 2 | 0.4 | 0.2 |
| 3 | Wall Bucky | 0 | 0 | 0 | 0 |
| 4 | Head pillow of CT CANON | 0.6 | 0 | 2.2 | 0.4 |
| 5 | RT side edge of patient table CT CANON | 0 | 0 | 0.4 | 0.4 |
| 6 | Center of patient table CT CANON | 1.6 | 0.4 | 8.8 | 10 |
| 7 | Head coil MRI 1 | 0.2 | 0.2 | 0 | 0 |
| 8 | Control panel for contrast media injector | 0 | 0 | 0 | 0 |
| 9 | Wall Bucky x-ray control panel | 0 | 0 | 0 | 0 |
| 10 | X-Ray cassette | 0 | 0 | 0 | 0.2 |
| 11 | Keyboard of CT CANON | 2 | 0.6 | 6 | 4 |
| 12 | RT side gantry control panel CT CANON | 0 | 0 | 0 | 0 |
| 13 | X-Ray cassette of primax X-Ray portable | 0 | 0 | 0 | 0 |
| 14 | LT side gantry control panel CT CANON | 0.4 | 1 | 0 | 0 |
| 15 | Primax X-Ray portable touch screen | 0 | 0.6 | 1 | 6 |
| 16 | CT CANON gantry | 0 | 0 | 0 | 0 |
| 17 | Hand of primax X-Ray portable | 0 | 0 | 0 | 0 |
| 18 | Anesthesia Machine | 0.4 | 0.2 | 0 | 0 |
| 19 | RT side of control panel of MRI gantry | 0.2 | 0 | 0 | 0 |
| 20 | Table of MRI control Room | 0 | 0 | 0.8 | 0.8 |
| 21 | LT side of control panel MRI gantry | 0 | 0 | 0 | 0 |
| 22 | Touch screen of X-Ray tube | 0 | 0 | 0 | 0 |
| 23 | Core of MRI gantry | 0 | 0 | 0 | 0 |
| 24 | Table of prep medication in X-Ray room | 0 | 0 | 0.8 | 0.2 |
| 25 | Keyboard of US for general use | 1.2 | 1.4 | 2.4 | 1.2 |
| 26 | Patient table od US for general use | 0.2 | 0 | 0 | 0 |
| 27 | Earplug of MRI | 0.2 | 0.2 | 0.8 | 0.4 |
| 28 | Center of patient table X-Ray | 0 | 0 | 0.2 | 0 |
| 29 | MRI surface coil 1 | 0.2 | 0 | 0.2 | 0.2 |
| 30 | RT side of MRI patient table | 4.8 | 1 | 0 | 0 |
| 31 | LT side of MRI patient table 1 | 0.4 | 0.6 | 20 | 20 |
| 32 | Trolley emergency in CT CANON room | 0.4 | 0 | 0.8 | 0.4 |
| 33 | keyboard in CT CANON control room | 3 | 0 | 0.6 | 1.6 |
| 34 | CT Siemens Keyboard control room | 4 | 8 | 6 | 6 |
| 35 | CT Siemens mouse control room | 0.8 | 0.4 | 0.6 | 0.4 |
| 36 | CT Siemens inside gantry | 0 | 0 | 0 | 0 |
| 37 | CT Siemens center of patient table | 0 | 0 | 0 | 0.4 |
| 38 | RT side edge of CT Siemens Patient table | 0.2 | 0 | 0.6 | 0.4 |
| 39 | CT Siemens head support | 0.8 | 0.6 | 0 | 0.8 |
| 40 | RT side CT Siemens gantry control panel | 0 | 0 | 0 | 0 |
| 41 | LT side CT Siemens gantry control panel | 0 | 0 | 0 | 0 |
| 42 | linear probe for interventional US | 0 | 0 | 0 | 0 |
| 43 | Curve linear probe for interventional US | 0 | 0 | 0 | 0 |
| 44 | The keyboard of interventional US | 1 | 0.6 | 0.2 | 0 |
| 45 | Touch screen of interventional US | 0 | 0.2 | 0.2 | 0 |
| 46 | Table of prep medication in CT Siemens room | 0 | 0 | 0 | 0 |
| 47 | Trolley emergency in CT Siemens room | 0.2 | 0 | 0 | 0 |
| 48 | Patient leg support of CT Siemens | 0.2 | 0 | 0.6 | 1.2 |
| 49 | Linear probe for US general use | 0 | 0 | 2.8 | 0.6 |
| 50 | Curve linear probe for US for general use | 0.2 | 0 | 0.4 | 0.4 |
| 51 | Large touch screen for US for general use | 0 | 0 | 12.8 | 0.4 |
| 52 | Table of US for general use in control room | 4.8 | 1.8 | 1.6 | 1.4 |
| 53 | Door of US for general use examination room | 0 | 0 | 0 | 0 |
| 54 | Small touch screen for US general use | 0.2 | 0 | 0.2 | 0.2 |
| 55 | Patient tabel tools in US for general use room | 0.8 | 0.2 | 0.6 | 0.4 |
| 56 | Hand of US for general use | 1 | 0.2 | 0.8 | 0.4 |
| 57 | Patient Head pillow in US for general use room | 0.6 | 0 | 0 | 0.6 |
| 58 | Mouse of US for general use in control room | 2.2 | 0.8 | 0.4 | 0.2 |
| 59 | keyboard in control room of US for general use | 3.4 | 0.6 | 1 | 0.2 |
| 60 | RT side of X-Ray patient table | 0.2 | 0 | 2.4 | 0.8 |
| 61 | LT side of X-Ray patient table | 0 | 0 | 0.2 | 0.4 |
| 62 | Keyboard in control room od X-Ray | 0 | 0.4 | 0.2 | 1 |
| 63 | Hand of carestearm portable | 8 | 0.2 | 0 | 0.8 |
| 64 | Carestream touchscreen portable | 0.2 | 0.2 | 5.2 | 5 |
| 65 | X-Ray cassette of carestream portable | 0 | 0 | 0.4 | 0 |
| 66 | LT side of patient table CT CANON | 8 | 8 | 1.6 | 1.2 |
| 67 | Touch screen of injector in CT CANON control room | 0.2 | 0 | 1.8 | 1.6 |
| 68 | Mouse of CT CANON | 0 | 0.2 | 0 | 0.6 |
| 69 | Mouse in CT CANON control room | 0 | 0 | 0 | 0 |
| 70 | Surface coil MRI 2 | 0 | 0 | 0 | 0 |
| 71 | Head coil MRI 2 | 0 | 0 | 0 | 0 |
| 72 | Knee coil | 2.4 | 3 | 0.6 | 0.4 |
| 73 | Center of MRI patient table 2 | 1.4 | 0 | 2.8 | 2.4 |
| 74 | RT side edge of MRI patient table 2 | 2.8 | 4.8 | 3 | 0.6 |
| 75 | LT side edge of MRI patient table 2 | 5.8 | 4.2 | 8.4 | 6 |
| 76 | LT side edge of CT Siemens patient table | 0.8 | 0 | 0.8 | 0 |
| 77 | Keyboard of X-Ray in control room | 3.2 | 3.4 | 6 | 8 |
| 78 | Mouse of X-Ray in control room | 0 | 0 | 1 | 0.8 |
| 79 | Patient leg support 1 | 2.2 | 2.4 | 0.4 | 0.4 |
| 80 | Patient leg support 2 MRI | 2 | 3.4 | 8 | 5.4 |
